# Tumor p38MAPK signaling enhances breast carcinoma vascularization and growth by promoting expression and deposition of pro-tumorigenic factors

**DOI:** 10.18632/oncotarget.18755

**Published:** 2017-06-28

**Authors:** Michelle Limoge, Alfiya Safina, Alexander M. Truskinovsky, Ieman Aljahdali, Justin Zonneville, Aleksandar Gruevski, Carlos L. Arteaga, Andrei V. Bakin

**Affiliations:** ^1^ Department of Cancer Genetics, Roswell Park Cancer Institute, Buffalo, New York, USA; ^2^ Department of Pathology, Roswell Park Cancer Institute, Buffalo, New York, USA; ^3^ Department of Cell Stress Biology, Roswell Park Cancer Institute, Buffalo, New York, USA; ^4^ Breast Cancer Research Program, Vanderbilt-Ingram Cancer Center, Vanderbilt University School of Medicine, Nashville, Tennessee, USA; ^5^ State University of New York at Buffalo, Department of Biological Sciences, Buffalo, New York, USA

**Keywords:** tumor microenvironment, breast cancer, p38MAPK, angiogenesis, fibronectin

## Abstract

The breast carcinoma microenvironment strikingly influences cancer progression and response to therapy. Various cell types in the carcinoma microenvironment show significant activity of p38 mitogen-activated protein kinase (MAPK), although the role of p38MAPK in breast cancer progression is still poorly understood. The present study examined the contribution of tumor p38MAPK to breast carcinoma microenvironment and metastatic capacity. Inactivation of p38MAPK signaling in metastatic breast carcinoma cells was achieved by forced expression of the kinase-inactive mutant of p38/MAPK14 (a dominant-negative p38, dn-p38). Disruption of tumor p38MAPK signaling reduced growth and metastases of breast carcinoma xenografts. Importantly, dn-p38 markedly decreased tumor blood-vessel density and lumen sizes. Mechanistic studies revealed that p38 controls expression of pro-angiogenic extracellular factors such as matrix protein Fibronectin and cytokines VEGFA, IL8, and HBEGF. Tumor-associated fibroblasts enhanced tumor growth and vasculature as well as increased expression of the pro-angiogenic factors. These effects were blunted by dn-p38. Metadata analysis showed elevated expression of p38 target genes in breast cancers and this was an unfavorable marker of disease recurrence and poor-outcome. Thus, our study demonstrates that tumor p38MAPK signaling promotes breast carcinoma growth, invasive and metastatic capacities. Importantly, p38 enhances carcinoma vascularization by facilitating expression and deposition of pro-angiogenic factors. These results argue that p38MAPK is a valuable target for anticancer therapy affecting tumor vasculature. Anti-p38 drugs may provide new therapeutic strategies against breast cancer, including metastatic disease.

## INTRODUCTION

Breast cancer is the second leading cause of cancer-related death among women in North America [1]. Despite advances in understanding tumor-intrinsic genomic alterations, it is still difficult to define patients with a higher risk of disease recurrence and metastasis. Growing evidence indicates that the tumor microenvironment (TME) is a significant contributing factor in cancer progression, recurrence, and response to therapy [2]. The TME includes blood vessels, stromal cells (fibroblasts, adipocytes) and infiltrating immune cells. The molecular pathways controlling the interaction between these major cellular components of the breast TME are still not fully understood [3, 4].

Triple-negative breast cancers are among the most aggressive and difficult to treat breast cancers with a significant presence of tumor-associated fibroblasts (TAFs) [3, 5]. Experimental evidence indicates that TAFs are the predominant cell type in the TME, which promote tumor infiltration by pro-tumorigenic myeloid immune cells such as macrophages, neutrophils and myeloid-derived suppressor cells (MDSCs) [6, 7]. In turn, these myeloid cells stimulate tumor vascularization and metastasis by secreting metalloproteinase MMP9/gelatinase-B [7–9], which increases recruitment of endothelial cells and pericytes [8, 10, 11]. Besides myeloid cells, MMP9 is also produced by breast carcinoma cells [12–14], and knockdown of MMP9 in carcinoma cells significantly reduces tumor vasculature [12]. Thus, all three cellular components of the breast TME can contribute to MMP9-driven tumor vascularization.

Breast carcinomas express elevated levels of cytokines such as transforming growth factor-β (TGF-β) and pro-inflammatory cytokines tumor necrosis factor (TNF) and interleukin 1β (IL-1β) [15–18]. These cytokines contribute to elevated expression of MMP9 in the TME [14, 19–21]. Ligation of these cytokines to cognate receptors activates mitogen-activated protein kinases (MAPK) such as p38MAPK, Jun-N-terminal kinase (JNK), and extracellular-regulated kinase (ERK) in various cell types of the breast TME [22]. In fact, significant activation of p38MAPK signaling is found in breast carcinomas and this has been linked to tumor progression [23]. Studies have shown that p38MAPK promotes breast carcinoma invasion and metastasis [24, 25] and may regulate MMP9 [26]. Among the four p38MAPK family members in the human genome, the p38-alpha kinase encoded by the *MAPK14* gene is ubiquitously expressed at high levels [22]. Recent studies with small-molecule inhibitors targeting p38-alpha/beta isoforms have shown promising results in preclinical studies and several anti-p38 drugs are under evaluation in clinical settings [27]. In preclinical animal models, p38-alpha/beta inhibitors have significantly reduced tumor xenograft growth and metastasis [25, 28]. Despite of these advancements, the role of tumor p38MAPK signaling in the breast carcinoma TME is still poorly understood.

The current study examined the contribution of tumor p38MAPK to breast carcinoma progression. We found that disruption of p38MAPK signaling in breast cancer cells by a kinase-inactive p38/MAPK14-AGF mutant (dn-p38) delayed tumor growth and formation of spontaneous metastasis in xenograft models. Immuno-histological analysis of tumor xenografts revealed a significant reduction in tumor vasculature in the dn-p38 xenografts. Studies of tumor-fibroblast interactions showed that fibroblasts enhanced tumor vasculature and growth of the control tumors, whereas this effect was lost in dn-p38 tumor xenografts. Mechanistic studies revealed that inactivation of p38 decreases expression of pro-angiogenic extracellular factors VEGFA, IL8, HBEGF and matrix protein Fibronectin. These findings indicate that tumor p38MAPK facilitates tumor vascularization by enhancing production and matrix-deposition of pro-angiogenic factors.

## RESULTS

### p38MAPK signaling contributes to tumor cell invasion and metastatic potential

**Figure 1:**
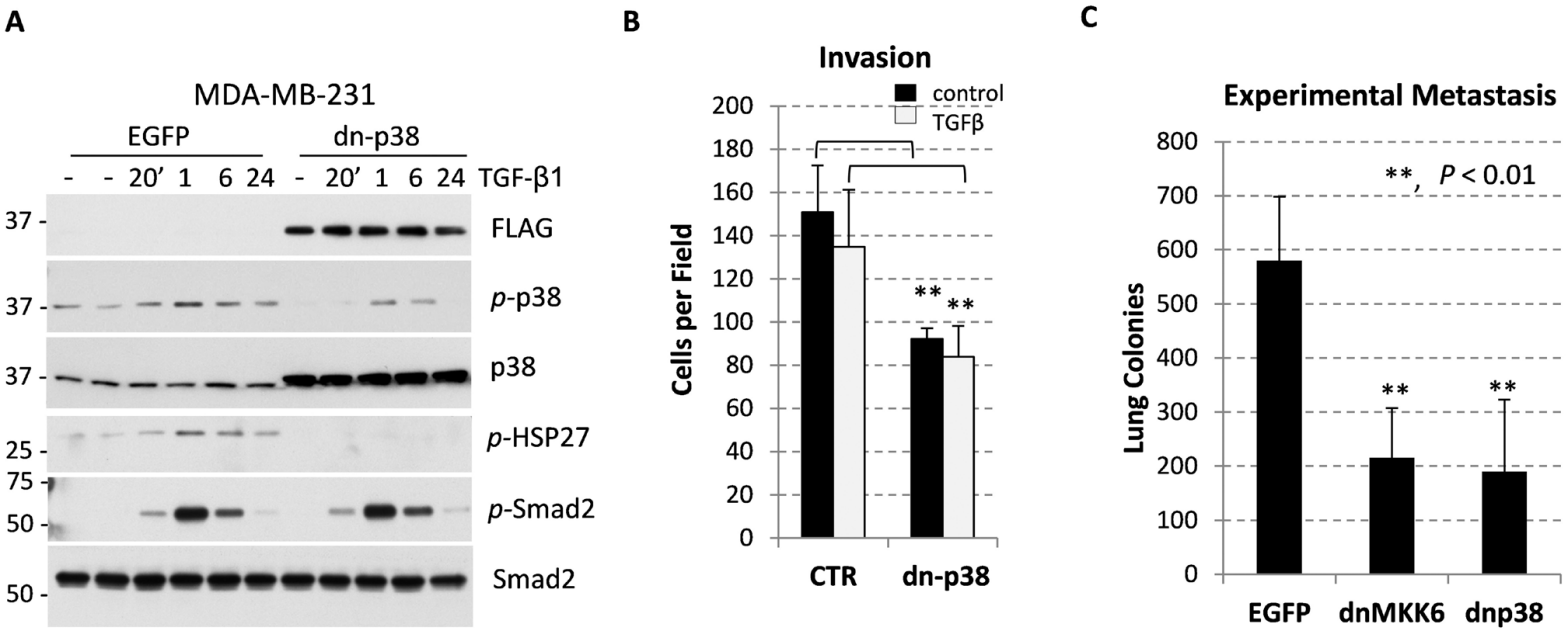
p38MAPK contributes to breast carcinoma invasion and metastasis **(A)** Immunoblotting of whole-cell lysates from breast cancer MDA-MB-231 cells transduced with empty-vector control (EGFP) or Flag-tagged p38MAPK-AGF (dn-p38). Cells were treated with 2 ng/mL TGF-β1 for the indicated times. **(B)** Invasion of MDA-MB-231 cells tested using Matrigel-covered transwells. Assays were done in triplicate and repeated at least twice. **(C)** Lung surface colonies of EGFP and dn-p38 MDA-MB-231 cells after tail-vein injection of tumor cells into female SCID mice, 6 mice/group). **, P<0.01

Systemic administration of selective p38-alpha/beta isoform inhibitors reduces both primary tumor growth and metastasis in breast carcinoma models [29, 30]. Here we asked whether inactivation of p38MAPK in breast cancer cells would influence tumor growth and the tumor microenvironment. Disruption of p38MAPK signaling was achieved by expression of a kinase-inactive p38MAPK-AGF mutant (a dominant-negative p38, dn-p38) in breast cancer MDA-MB-231 cell line, established from a patient with metastatic triple-negative breast cancer (TNBC). Dn-p38 strategy better mimics a treatment with kinase inhibitors compared to a depletion strategy using RNA interference or gene-disruption approaches. Tumor cells were infected with empty-vector control and dn-p38 retroviruses, which also encoded enhanced green-fluorescence protein (EGFP) translated from an internal ribosome entry site (IRES) [24]. EGFP-positive cell populations were selected for further studies. Immunoblot analysis of EGFP-positive cells revealed expression of FLAG-tagged dn-p38 protein comparable with endogenous p38MAPK (Figure 1A). Dn-p38 effectively blocked phosphorylation of HSP27, a well-known p38 target, in response to TGF-β1 treatment (Figure 1A). Dn-p38 also reduced levels of active phosphorylated p38MAPK but did not affect phosphorylation of Smad2, as expected (Figure 1A). Dn-p38 did not affect phosphorylation of ERK (Supplementary Figure 1). These findings argue that dn-p38 selectively inactivated p38MAPK signaling.

Next, we assessed invasive and metastatic potential of EGFP- and dn-p38 cell populations. Dn-p38 reduced invasion *in vitro* and lung metastasis in a tail-vein experimental metastasis model (Figure 1B, 1C). The latter finding was further validated using MDA-MB-231 cells expressing a kinase-inactive MKK6-AL mutant form of MKK6 (dn-MKK6) [24], a p38MAPK activating kinase (Figure 1C). Thus, p38MAPK signaling contributes to the invasive and metastatic capacities of breast carcinoma cells.

### p38MAPK signaling in primary tumor growth and metastasis of breast carcinomas

**Figure 2:**
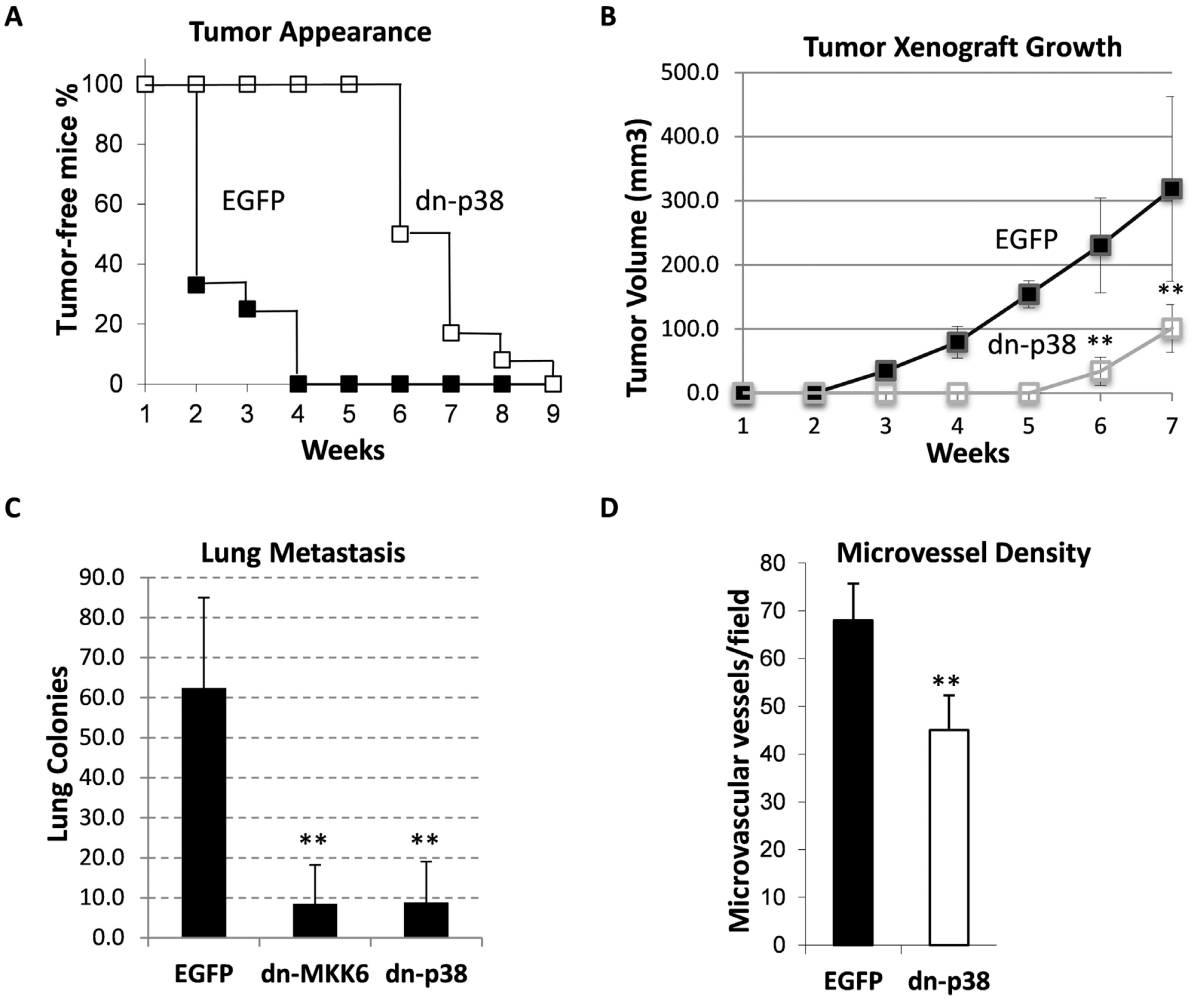
Tumor growth and lung metastasis of MDA-MB-231 cell xenografts in the orthotopic model **(A)** Appearance of palpable tumors following injection of breast cancer MDA-MB-231 EGFP and dn-p38 cells into the mammary fat pad of SCID mice. **(B)** Tumor volume of the orthotopic xenografts of EGFP and dn-p38 MDA-MB-231 cells in female SCID mice, 6 mice/group. **(C)** Lung-surface colonies of tumor cells in the orthotopic xenograft model, 6 mice/group. **(D)** Microvessel density measured using CD31 staining of tumor sections in six fields for each tumor section (5 tumors per group) and presented as a mean number per field (0.2 mm^2^). **, P<0.01

We then examined the effect of dn-p38 on primary tumor growth and metastasis using an orthotopic xenograft model. Empty-vector control and dn-p38-MDA-MB-231 cells were placed in the mammary fat-pad of female SCID mice. The appearance of palpable tumors in the dn-p38 group was delayed by nearly four weeks (Figure 2A). Primary tumor growth was reduced in the dn-p38 group (Figure 2B) although dn-p38 did not reduce cell growth in cell-culture assays (Supplementary Figure 2). To evaluate metastasis, primary tumors were surgically removed at a 1-cm diameter and lung-surface metastatic colonies were scored using India ink staining after 30-35 days. The number of metastatic colonies was reduced in the dn-p38 group by nearly 6 times compared to the empty-vector control group (Figure 2C). A similar result was obtained in the dn-MKK6 group (Figure 2C). Next, we assessed tumor blood vessels using CD31/PECAM staining of histological tumor sections. Microscopic analysis revealed a significant reduction in the blood microvessel density in dn-p38 tumors (Figure 2D), suggesting a defect in tumor vascularization. Together, these results indicate that p38MAPK signaling contributes to breast carcinoma growth and metastasis, in part, by facilitating tumor cell invasiveness and tumor vascularization.

### Tumor-associated fibroblasts enhance tumor growth and this is blocked by tumor dn-p38

**Figure 3:**
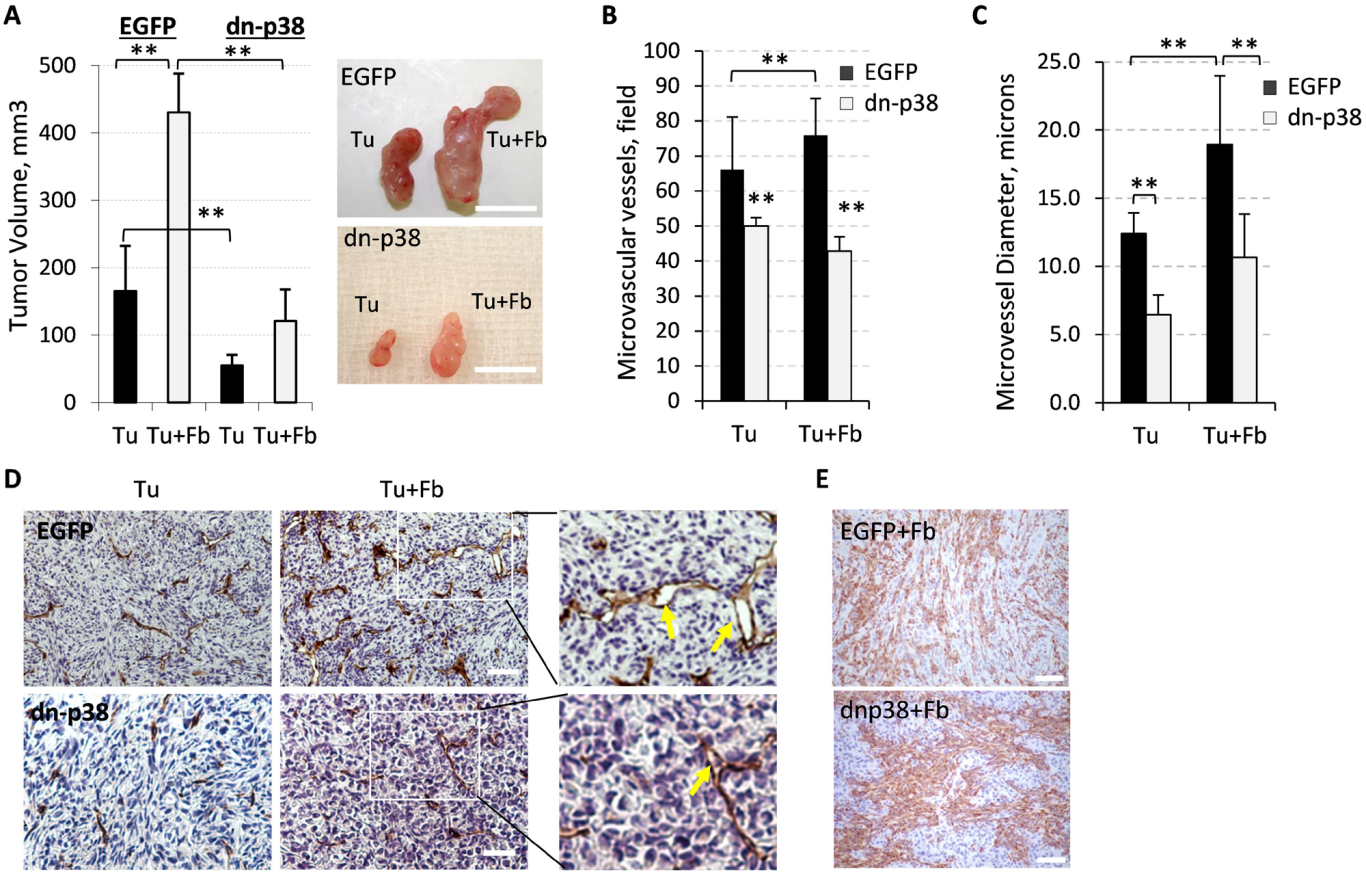
Fibroblast-enhanced tumor growth and vasculature depend on tumor p38MAPK **(A)** Graphs show tumor volumes at the endpoint of the xenograft study with empty-vector control (EGFP) and dn-p38 MDA-MB-231 breast carcinoma cells alone (Tu) or in combination with 208F fibroblasts (Tu+Fb). Images show tumors excised at the endpoint of the study. **, P<0.01. **(B)** Quantification of the microvessel density was done using CD31 staining of tumor sections in six fields for each tumor section (5 tumors per group) and presented as a mean number per field (0.2 mm^2^). **(C)** Blood-vessel diameters in tumor sections were measured at 400× magnification on slides immune-stained for CD31. **, P<0.01. **(D)** CD31 staining (blood vessels) of tumor xenograft sections of breast carcinoma MDA-MB-231 EGFP or dn-p38 cells alone (Tu) or in combination with fibroblasts (Tu+Fb); arrows mark vessels with large lumen. Enlarged images of boxed areas are shown in the right panels. **(E)** Staining of fibroblasts with an antibody to rPH, prolyl 4-hydroxylase. Images were taken at 400x magnification, bar size, 100μm.

Recent studies suggest that tumor-associated fibroblasts enhance tumor growth in part by enhancing tumor angiogenesis [31] and p38MAPK may contribute to some of these effects [28]. We tested whether supplementation of fibroblasts would enhance growth of dn-p38-expressing tumors. Co-injection of empty-vector control MDA-MB-231 cells together with fibroblasts increased tumor growth compared to tumor cells alone, Tu+Fb *vs* Tu (Figure 3A). Fibroblasts alone did not form tumors (data not shown). The growth of tumor cell xenografts alone or in combination with fibroblasts was still markedly impaired in the dn-p38 groups compared to the control groups (Figure 3A). The mean value of tumor volumes at the endpoint of the study was nearly 4 times lower in the dn-p38 group compared to the control (Figure 3A).

Evaluation of the tumor vasculature using CD31 staining showed that fibroblasts enhanced the microvessel density by about 15% in EGFP-tumors (Figure 3B). In dn-p38 tumors the blood vessel density was significantly reduced in both tumor cell alone and tumor-fibroblast combination groups (Figure 3B). Microscopic examination of CD31-stained tissues revealed that lumen sizes of the microvessels were increased in the tumor-fibroblast group compared to the tumor cell alone group and this effect was negated by dn-p38 (Figure 3C-3D). To assess whether this defect is associated with a reduction in fibroblasts, we stained tumor sections with antibodies to a fibroblast-specific marker prolyl 4-hydroxylase, rPH. This analysis showed that dn-p38 did not affect the presence of fibroblasts in tumors (Figure 3E). Together, these results indicate that tumor p38MAPK is critical for tumor vascularization. Fibroblasts enhanced tumor growth in part by increasing the blood vessel density and lumen size. These effects were largely negated by inactivation of tumor p38MAPK signaling.

### The effect of p38MAPK signaling on expression of pro-angiogenic factors

**Figure 4:**
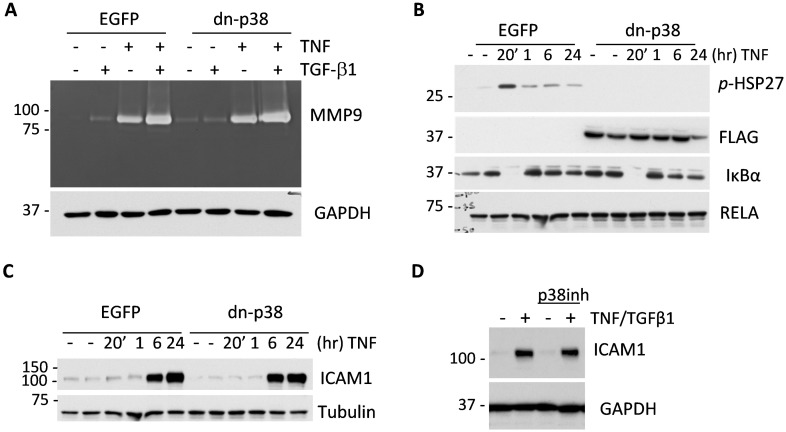
Disruption of p38MAPK signaling does not reduce expression of MMP9 and ICAM1 by tumor cells **(A)** Top panel shows gelatin zymography of 48-hour conditioned media from empty-vector control (EGFP) and dn-p38 MDA-MB-231 cells treated with 2 ng/mL TGF-β1 or 10 ng/mL TNF or their combination. Bottom panel shows immunoblotting of GAPDH, a loading control, in whole-cell lysates. **(B)** Immunoblot analysis of signaling markers in response to TNF in EGFP and dn-p38 MDA-MB-231 cells treated with 10 ng/mL TNF for the indicated times. **(C)** Immunoblotting of ICAM1 in whole-cell lysates from EGFP and dn-p38 MDA-MB-231 cells treated with 10 ng/mL TNF for the indicated times. Tubulin is a loading control. **(D)** Immunoblots of ICAM1 and GAPDH in lysates of MDA-MB-231 cells treated with 2 ng/mL TGF-β1 and 10 ng/mL TNF ± 5 μM SB202190, a p38MAPK inhibitor, for 24 hours.

**Figure 5:**
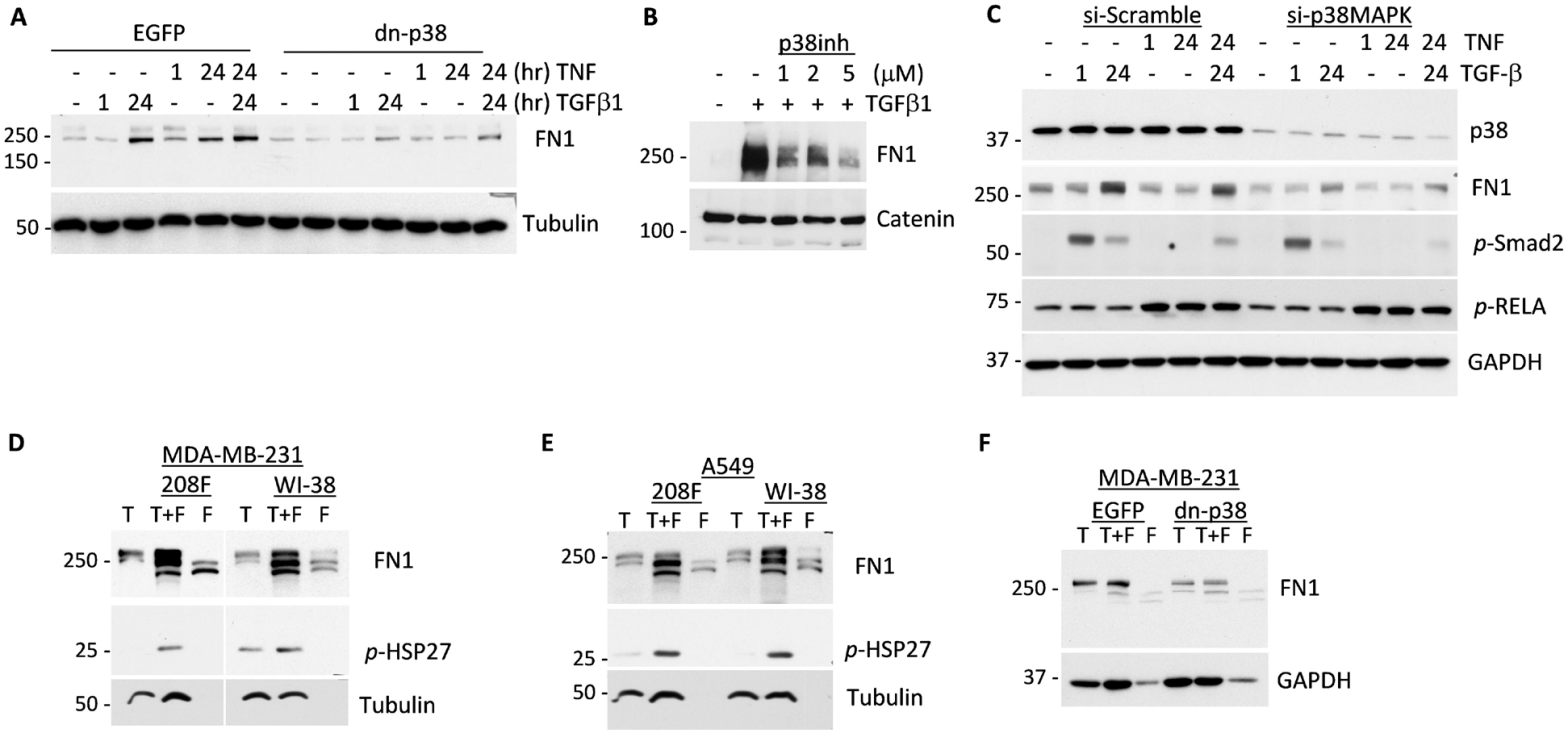
p38MAPK signaling contributes to expression of Fibronectin in response to cytokines and tumor-fibroblast interactions **(A)** Immunoblotting of Fibronectin and tubulin, a loading control, in whole-cell lysates from empty-vector control (EGFP) and dn-p38 MDA-MB-231 tumor cells treated with 2 ng/mL TGF-β1 or 10 ng/mL TNF or their combination for the indicated times. **(B)** Immunoblot analysis of Fibronectin and α-catenin, a loading control, in lysates of tumor cells treated with SB202190, a p38 inhibitor, and 2 ng/mL TGF-β1 for 24 hours. **(C)** Immunoblots of Fibronectin, p38, p-Smad2, p-RELA and GAPDH, a loading control, in lysates of MDA-MB-231 cells transfected with Scramble-control or siRNA to p38-alpha and then treated with 2 ng/mL TGF-β1 or 10 ng/mL TNF or their combination for the indicated times. **(D-E)** Immunoblotting of Fibronectin, phospho-HSP27 and tubulin, a loading control, in lysates from co-cultures (T+F) of MDA-MB-231 and A549 cancer cells (T) with 208F and WI-38 fibroblasts **(F)** for 72 hours. **(F)** Immunoblots of Fibronectin and GAPDH, a loading control, in lysates from co-cultures (T+F) of MDA-MB-231 EGFP or dn-p38 cells (T) and 208F fibroblasts **(F)** incubated for 48 hours.

To address the mechanism by which p38MAPK enhances tumor angiogenesis we examined the expression of MMP9 and ICAM1. Several studies have shown a critical role of MMP9 in tumor angiogenesis [8, 12] and have linked ICAM1 to intra-tumor microvessel density [32]. ICAM1 is also considered a target in triple-negative breast cancer [33]. Assessment of MMP9 by in-gel gelatin-zymography assays showed that TNF and TGF-β cytokines induced secretion of MMP9 by breast cancer cells and this was further enhanced by co-treatment with both cytokines together (Figure 4A). MMP9 regulation was not altered by dn-p38 (Figure 4A). We then examined whether dn-p38 affects TNF signaling. TNF induced phosphorylation of HSP27 and a rapid degradation of IκBα, releasing RELA which up-regulates expression of IκBα by 60 min (Figure 4B), setting up a feedback shutoff of NF-κB signaling [34]. Dn-p38 blocked phosphorylation of HSP27 but did not affect TAK1-IκBα signaling in response to TNF (Figure 5B). Assessment of ICAM1 expression showed that TNF strongly stimulated expression of ICAM1 protein in both empty-vector control and dn-p38 cells (Figure 4C). This response was verified using a selective p38 inhibitor, SB202190. As expected, inhibition of p38 reduced phosphorylation of HSP27 in response to TGF-β1 (Supplementary Figure 3). However, the p38 inhibitor did not block induction of ICAM1 (Figure 4D). These results demonstrate that inhibition of p38MAPK signaling does not block basal and TNF-induced expression of ICAM1 or induction of MMP9 by TGF-β and TNF cytokines.

### p38MAPK signaling contributes to expression of Fibronectin and cytokines

We then examined whether dn-p38 alters expression of pro-angiogenic factors such as extracellular matrix protein Fibronectin, an anchor of VEGFA, and pro-angiogenic cytokines including VEGFA and IL8/CXCL8. In EGFP-control MDA-MB-231 cells, treatment with TGF-β1 and TNF cytokines increased protein levels of Fibronectin (Figure 5A). In dn-p38 cells, up-regulation of Fibronectin was blocked (Figure 5A). A p38MAPK inhibitor SB202190 also suppressed induction of Fibronectin in response to TGF-β1 (Figure 5B). In control, SB202190 blocked phosphorylation of HSP27, a marker of p38 signaling, while did not reduce phosphorylation of Smad2 (Supplementary Figure 3). To validate these findings, p38MAPK (alpha) was suppressed using siRNA in MDA-MB-231 cells (Figure 5C). Depletion of p38 markedly reduced basal and cytokine-stimulated levels of Fibronectin (Figure 5C).

We next examined whether co-culturing of tumor and fibroblast cells would regulate the p38MAPK-HSP27 axis and Fibronectin expression. In co-cultures of MDA-MB-231 cells with 208F or WI-38 fibroblasts, levels of Fibronectin and phosphorylation of HSP27 were significantly elevated compared to individual cell cultures alone (Figure 5D). Similar results were obtained in fibroblast co-cultures with lung carcinoma A549 cells (Figure 5E). To validate whether tumor p38MAPK is responsible for the up-regulation of Fibronectin levels, we examined co-cultures with dn-p38 cells. Fibronectin levels were elevated in co-cultures of MDA-MB-231-EGFP and 208F cells, whereas this response was decreased in the MDA-MB-231-dn-p38 co-cultures (Figure 5F). Together these results strongly support a critical role of p38MAPK in induction of Fibronectin levels in response to TGF-β/TNF cytokines and by tumor-fibroblast co-cultures.

**Figure 6:**
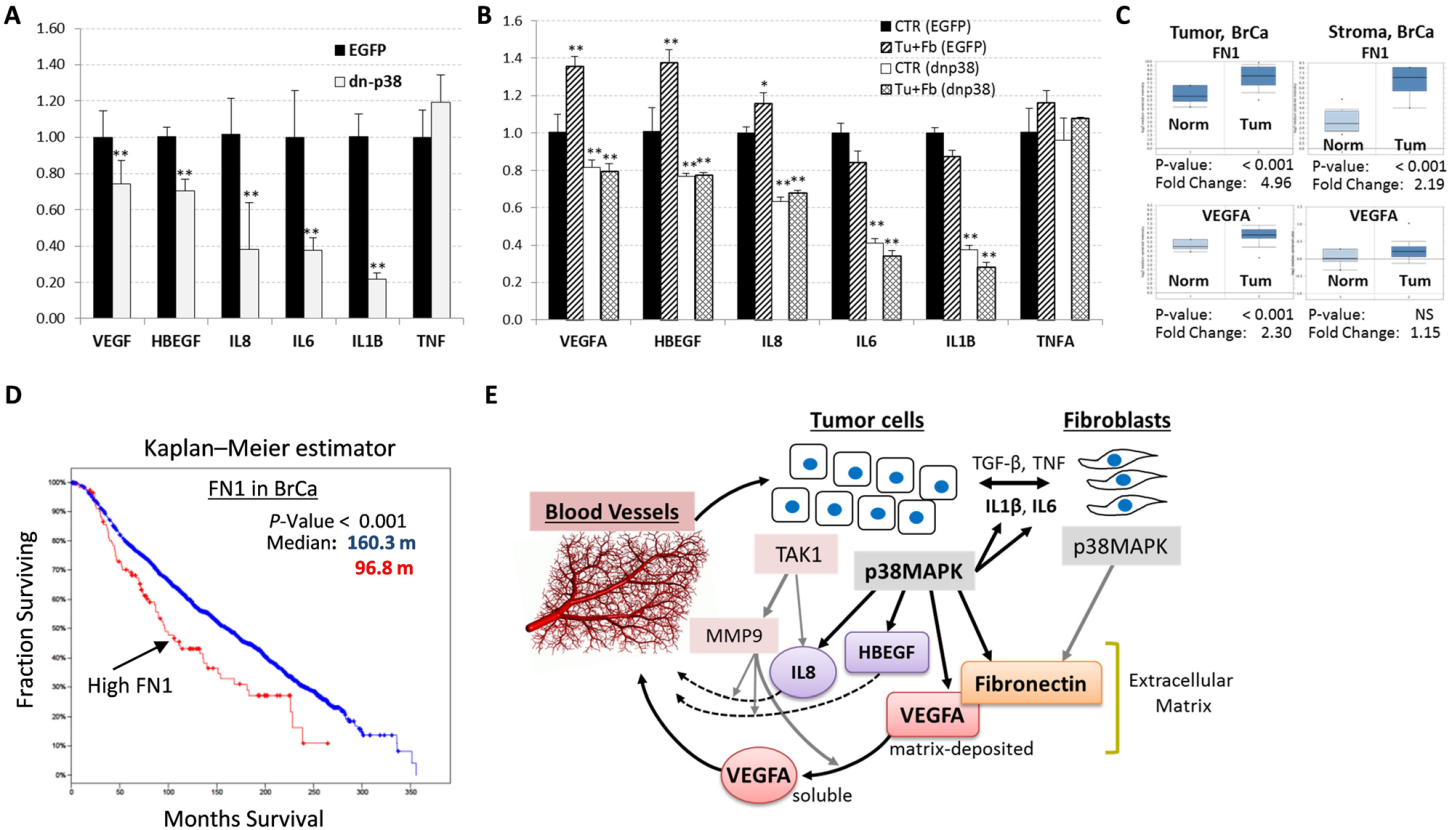
Levels of p38MAPK targets are elevated in human breast cancer **(A)** qRT-PCR analysis of pro-angiogenic cytokine mRNA levels in empty-vector control (EGFP) and dn-p38 MDA-MB-231 cells. **(B)** qRT-PCR of pro-angiogenic cytokine mRNA levels in EGFP and dn-p38 MDA-MB-231 cells individually cultured or in co-cultures with 208F fibroblasts for 48 hours. **(C)** Expression of Fibronectin (FN1) and VEGFA in the tumor and stromal compartments of human breast carcinomas obtained using data from the Richardson Breast Study (tumor), and the Finak Breast Study (stroma). **(D)** Kaplan-Meier survival estimation of the overall survival of breast cancer patients and Fibronectin levels using the Breast Cancer METABRIC dataset [35]. **(E)** A model of the p38MAPK role in the regulation of tumor angiogenesis in breast carcinomas. Crosstalk of tumor and fibroblast cells increases cytokine signaling *via* p38MAPK and TAK1/MAP3K7. p38MAPK promotes expression of Fibronectin, an extracellular matrix protein as well as pro-angiogenic cytokines including VEGFA, IL8 and HBEGF. TAK1 controls expression of MMP9 which releases matrix-bound VEGFA and activates IL8. Pro-angiogenic cytokines stimulate tumor vascularization thereby enhancing tumor growth. *, P<0.05, **, P<0.01.

To explore whether p38MAPK promotes expression of pro-angiogenic cytokines, we measured their mRNA levels in control (EGFP) and dn-p38 cells. Quantitative RT-PCR assays revealed that mRNA levels of VEGFA, IL8, HBEGF, IL1B, and IL6 were significantly reduced in dn-p38-MDA-MB-231 cells compared to EGFP-control cells (Figure 6A). Furthermore, mRNA levels of VEGFA, IL8, and HBEGF were elevated in tumor-fibroblast co-cultures compared to individual cell cultures (Figure 6B). Levels of these cytokines were significantly reduced in dn-p38 cells and their co-cultures with fibroblasts (Figure 6B). These findings demonstrate that p38MAPK contributes to the expression of pro-angiogenic cytokines by tumor cells alone and in co-cultures with fibroblasts.

### Expression of p38MAPK targets and breast cancer

To determine whether levels of p38MAPK-dependent targets are altered in breast cancer, we assessed mRNA levels of p38 targets in human breast cancer genomic datasets. Analysis using the Oncomine research tool revealed that mRNA levels of Fibronectin (FN1) and VEGFA are significantly elevated in the tumor and stromal compartments of breast carcinomas (Figure 6C). A high level of Fibronectin also correlated with disease recurrence within 5 years of follow-up (Supplementary Figure 4). Kaplan-Meier survival estimation using the Breast Cancer METABRIC dataset [35] showed that high levels of Fibronectin is associated with shorter survival of breast cancer patients (Figure 6D). A similar result was obtained in the analysis of both FN1 and VEGFA (data not shown). Thus, expression levels of p38MAPK targets FN1 and VEGFA are elevated in breast cancers and a high level of these factors is an unfavorable marker of disease recurrence and poor-outcome.

## DISCUSSION

The current study found that p38MAPK signaling in tumor cells promotes breast carcinoma growth and metastasis by altering the tumor microenvironment (TME). Inactivation of p38MAPK signaling in breast carcinoma cells reduced growth and spontaneous metastasis of tumor xenografts. Immuno-histological analysis revealed a significant reduction in vasculature of the dn-p38 tumors. Studies of tumor-fibroblast interactions showed that fibroblasts enhanced tumor vasculature and growth, increasing the microvessel density and lumen size of blood vessels. These effects were negated by dn-p38. Mechanistic studies showed that p38 contributes to expression of extracellular factors stimulating blood vessel formation such as Fibronectin and pro-angiogenic cytokines, including VEGFA. Thus, our study revealed a previously unappreciated role of tumor p38MAPK signaling in promoting tumor angiogenesis.

Our data show that p38MAPK signaling in breast carcinoma cells enhances tumor vascularization and metastasis (Figures 2-3). p38MAPK controls expression of Fibronectin and several pro-angiogenic cytokines including HBEGF, IL6 and VEGFA (Figures 5-6). The study ruled out involvement of p38MAPK in expression of pro-tumorigenic factors MMP9 and ICAM1, and TNF-stimulated NF-κB signaling (Figure 4). These findings are consistent with reports on a critical role of the TAK1-NF-κB module in MMP9 expression [31], and NF-κB in the regulation of ICAM1 in breast cancer cells [36]. In addition to pro-angiogenesis activity, p38 also contributes to tumor invasion (Figure 1B). This result is consistent with several previous reports showing a critical role of p38 in epithelial-mesenchymal transition and invasion [25, 26, 28, 37–41]. In some cell systems, however, depletion of p38 may enhance tumor invasion [42]. This latter study seemingly contradicts multiple aforementioned reports and needs further validation.

We were puzzled by the strong impact of p38 inactivation on tumor vasculature even though MMP9 was not affected. Tumor vascularization involves a release of matrix-deposited VEGFA by MMP9 produced by various cells in the TME [7-9, 31]. Potentially, p38 may control expression or deposition of these factors. Investigating along these lines, we found that p38 contributes to expression of Fibronectin by tumor cells (Figure 5). Fibronectin is among the key extracellular matrix (ECM) proteins which anchor VEGFA in the TME and promote angiogenesis [43, 44]. We found that Fibronectin is expressed by both tumor and fibroblast cells (Figure 5). Furthermore, Fibronectin and VEGFA levels were enhanced in tumor-fibroblast co-cultures in a p38MAPK-dependent manner, as genetic or pharmacological inhibition of p38 reduced Fibronectin levels (Figure 5-6). In addition, our data indicate that p38 controls expression of HBEGF, IL8/CXCL8, and IL6 (Figure 6A, 6B). HBEGF may contribute to tumor angiogenesis [45–47] and is produced as a cell membrane-anchored protein that can be released in a soluble form by MMP9 [47]. HBEGF may also enhance breast cancer growth and invasiveness [48]. IL8/CXCL8 promotes angiogenesis and can be activated by MMP9 [49]. Thus, p38MAPK controls expression and deposition of pro-angiogenic and pro-tumorigenic factors enhancing carcinoma growth and metastasis (Figure 6E).

Meta-analysis revealed that human breast carcinomas express elevated levels of Fibronectin (FN1) and VEGFA compared to normal tissues (Figure 6C). High levels of Fibronectin correlate with poor prognosis and a shorter survival due to breast cancer related death (Figure 6D), suggesting that p38MAPK is a potential target for anticancer therapy. This idea is further supported by recent pre-clinical studies with a pharmacological inhibitor of p38-alpha/beta isoforms [30]. Nonetheless, clinical application of anti-p38 drugs should proceed with caution given the reported tumor suppressor activities of p38/MAPK14 [50].

In summary, the current study revealed a previously unappreciated role of tumor p38MAPK in promoting tumor vasculature by enhancing expression of pro-angiogenic and pro-tumorigenic factors (Figure 6E). In the breast TME, tumor and stromal cells stimulate cytokine-mediated p38MAPK signaling that increases expression of pro-angiogenic and pro-invasive factors such as VEGFA, IL8, IL6, HBEGF, and Fibronectin. VEGFA is anchored by Fibronectin in the extracellular matrix, and then soluble VEGFA is released from matrix by metalloprotease MMP9 produced by tumor or infiltrating myeloid cells *via* TAK1 signaling. MMP9 can also activate IL8 or membrane-anchored HBEGF. Active VEGFA, IL8 and HBEGF then stimulate tumor vasculature acting upon endothelial cells or pericytes. In addition, HBEGF and IL6 can promote tumor growth/invasion acting on tumor cells. Our findings suggest that p38MAPK is a potential target for anticancer therapy inhibiting tumor vasculature and invasion stimulated by tumor-associated stroma. Anti-p38 drugs should provide new therapeutic options for treatment of breast cancer, including metastatic disease.

## MATERIALS AND METHODS

### Cytokines, antibodies and other reagents

Recombinant human TGF-β1 (Cat# 240-B/CF) was obtained from R&D Systems (Minneapolis, MN); recombinant human TNFα (Cat# CYT-223) was from ProSpec-Tany TechnoGene Ltd (Rehovot, Isreal). Antibodies for: GAPDH (Cat# sc-25778), p38MAPK(sc-81621) and IκBα (Cat# sc-371) were from Santa Cruz Biotechnology, Inc. (Santa Cruz, CA); phospho-Smad2 (Cat# 3108), phospho-HSP27 (Cat# 2401), phospho-p38 (Cat# 9211), RELA/p65 (Cat# 8242) and ICAM1 (Cat# 4915) were from Cell Signaling Technology (Danvers, MA); α-Tubulin (Cat# T6074), α-Catenin (Cat# C2081) and FLAG (Cat# F3165) were from Sigma-Aldrich (St. Louis, MO); FN1 (Cat# 610077) and Smad2 (Cat# 610842) were from BD Biosciences (San Jose, CA). Goat anti-Rabbit IgG (H+L)-Horseradish Peroxidase (HRP) (Cat# 170-6515) and goat anti-Mouse IgG (H+L)-HRP (CAT# 170-6516) secondary antibodies were from BIO-RAD Laboratories (Hercules, CA). Inhibitor of p38, SB202190 (Cat# 559388) was obtained from Calbiochem (EMD Millipore; Billerica, MA). Retroviral constructs encoding EGFP, FLAG-p38MAPK-AGF and HA-MKK6-AL are described in [24]. Short interfering RNA (siRNA) to human p38-alpha (MAPK14; Cat # 1299001; VHS40416; sequence CCAAAUUCUCCGAGGUCUAAAGUAU) was from Thermo Fisher Scientific (Waltham, MA).

### Cell culture

Human metastatic breast carcinoma cell line MDA-MB-231, human lung carcinoma cell line A549, rat embryonic fibroblast cell line 208F and human embryonic fibroblast cell line WI-38 were obtained from American Tissue Culture Collection (ATCC) (Manassas, VA) and cultured as recommended by ATCC. MDA-MB-231-EGFP, MDA-MB-231-p38MAPK-AGF, and MDA-MB-231-MKK6-AL cells expressing EGFP, Flag-tagged kinase-inactive p38MAPK-AGF (dn-p38), or HA-tagged kinase-inactive MKK6-AL (dn-MKK6) were generated by retroviral transduction and are previously described elsewhere [20, 24].

### Immunoblot analysis

A detailed description of immunoblotting has been reported elsewhere [20, 21]. Briefly, whole-cell lysates were collected using NP40 Lysis Buffer (0.88% NP-40, 132 mM NaCl, 44 mM Hepes, 8.8 mM NaF) supplemented with 2 mM sodium orthovanadate, 1 mM PMSF and 1X Protease Inhibitor Cocktail (Cat# 11836153001; Roche; Basel, Switzerland). Prior to lysis, cells were grown to 70-80% confluency and, if necessary, treated with 2 ng/mL TGF-β1 and/or 10 ng/mL TNFα for indicated times. Inhibitors were added 1 hour prior to cytokine treatment. Protein concentrations were measured using the Bio-Rad DC Protein Assay according to the manufacturer’s instructions. Proteins were resolved using 10% SDS-PAGE and transferred to nitrocellulose membranes (Cat# 162-0112; BIO-RAD). Transfer was validated by Ponceau S staining. Membranes were blocked with 5% milk for 1 hour at room temperature (RT) then incubated with the primary antibody in 5% milk overnight at 4°C. After washing, membranes were incubated with secondary antibodies in 5% milk for 1 hour at RT. Protein bands were visualized using ECL chemiluminescent reagent (Cat# 32209; Pierce).

### Matrigel invasion assay

Cells were mildly trypsinized and washed twice in IMEM with 0.1% BSA. Cells were seeded 1 × 10^5^ in the upper Matrigel-coated chamber (Cat# 354480; Calbiochem). The lower chamber was filled with 0.6 mL IMEM containing 0.1% BSA. TGFβ1 (1 ng/mL) was added to the lower chamber. After 20 hr of incubation, the non-migrating cells in the upper chamber were wiped away and migrating cells present on the lower surface of the insert were stained with Diff-Quik Stain (Biochemical Sciences Inc.; Swedesboro, NJ). Invading cells were counted from five random fields in three wells. Experiments were repeated at least two times.

### Short interference RNA

Cells were transfected with RNA duplexes using Lipofectamine 2000 (Cat# 11668027; Invitrogen, Thermo Fisher Scientific; Waltham, MA) following the manufacturer’s protocol. Cells were seeded and grown in the absence or presence of 2 ng/mL TGF-β1 and/or 10 ng/mL TNFα followed by immunoblotting.

### Animal housing

Female SCID/CB17 mice, 6 weeks of age, were obtained from a colony of SCID/CB17 mice bred and maintained at the Department of Laboratory Animal Resources (DLAR) facility at RPCI. Animals were kept 5 mice per cage in microinsulator units and provided with food and water *ad libitum* according to a protocol approved by the Institute Animal Care and Use Committee (IACUC) at RPCI. The facility has been certified by the American Association for Accreditation of Laboratory Animal Care and in accordance with current regulation and standards of the US Department of Agriculture and the US Department of Health and Human Services.

### Animal studies using a tail-vein injection model

Exponentially growing breast cancer cells (2.5 × 10^6^) in 0.2 mL of sterile Hank’s buffered salt solution were injected using a 28G needle into the tail-vein of 8 week old female SCID mice. Mice were sacrificed after 4 weeks. Lungs were perfused with India black ink and tumor colonies on lung surfaces were counted as described in [12, 51].

### Orthotopic xenograft model

Exponentially growing breast cancer cells (1 × 10^6^) in 0.1 mL Hank’s buffered salt solution were inoculated into the surgically exposed mammary fat pad of 7 to 8 week old female SCID mice. The growth of primary tumors was monitored by measuring tumor diameters with electronic calipers every 3-4 days continuously from the third week after injection. Volumes were calculated using the formula (length) × (width)^2^/2. Primary tumors were removed at 1 cm diameter, typically 30-35 days after appearance of palpable tumors. After 4-5 weeks, the mice were sacrificed and lungs, bones, spleens and livers were collected for histological analysis at the RPCI Pathology Core.

### Animal studies using a subcutaneous xenograft model

Exponentially growing breast cancer cells (1.5 × 10^6^) in 0.1 mL 50% sterile phosphate buffered solution (PBS): 50% reduced growth factor basement membrane extract type 2 (BME) were injected with a 27G needle into the left flank of 8 week old female SCID mice. Breast cancer cells (1.5 × 10^6^) mixed in a 3:1 ratio with exponentially growing fibroblast cells (0.5 × 10^6^) in 0.1 mL 50% PBS: 50% BME were injected into the right flank of the same 8 week old female SCID mice. Primary tumor growth was monitored by measuring tumor diameters with electronic calipers every 2-3 days after the appearance of palpable tumors. Volumes were calculated using the formula (length) × (width)^2^/2. After 9 weeks, mice were sacrificed and tumors were collected for histological analysis at the RPCI Pathology Core Facility.

### Immunohistochemistry

Tumor sections were fixed immediately after excision in 10% (v/v) formalin or, for CD31 staining, Zinc Fixative (Cat# 550523; BD Biosciences, NJ) before embedding in paraffin. Before immunostaining, conventional H&E-stained sections were prepared for general histopathological evaluation. For CD31 staining, rat anti-mouse primary antibody to CD31 (Cat#550274, BD Biosciences) and biotinylated secondary anti-rat antibody (BD Biosciences) were used as described in [12]. Analysis of microvessel density was performed as described in [52]. Briefly, tumor sections were scanned at 100× magnification for areas containing the highest number of discrete CD31-positive microvessels (microvessel hot spots). Necrotic and immediately adjacent areas where microvessels are sparse were excluded from counting. Rat fibroblasts were detected by staining tumor sections with an antibody to the rat fibroblast specific marker prolyl 4-hydroxylase (6-9H6) (NBP2-33342; Novus Biologicals; Littleton, CO). CD31-positive (brown stain) vessels were counted at 400× magnification in 8 fields of each tumor section. Results were presented as mean number of microvessels/field (0.2 mm^2^) ± standard deviation. The luminal size of blood vessels was evaluated on slides immunostained for CD31 using a DP26 Olympus microscope camera attached to a BX46 Olympus microscope and cellSens Standard 1.11 imaging software by Olympus. At low magnification, the areas with the largest blood vessels in each case were identified and the diameters of the five largest vessels in each of five microscopic fields were measured at 400× magnification in five tumors from each group. The results were presented as the mean number of diameters in each group ± standard deviation.

### Gelatin zymography assay

Conditioned media from cells untreated or treated with cytokines and/or inhibitors in serum-free IMEM or DMEM was collected, centrifuged and concentrated as needed. SDS-PAGE gels were co-polymerized with gelatin at a final concentration of 1 mg/mL. After electrophoresis (120V, ∼1.5 hours), the gels were renatured in 2.5% Triton X-100 and incubated in development buffer (0.05M TrisHCl pH 7.4, 5mM CaCl_2_, 0.2M NaCl, 0.02% NaN_3_) at 37°C with agitation for 18 hours. Gels were stained with Coomassie solution (0.5% (w/v) Coomassie Blue R250, 5% (v/v) methanol, 10% (v/v) acetic acid) for 2 hours followed by incubation in destaining solution (20% (v/v) methanol, 10% (v/v) acetic acid). Gelatinase activity is seen as a transparent band on a blue background.

### *In vitro* co-culture

Tumor cells were seeded 4.5 × 10^5^ cells per well in a 6-well plate individually or in combination with fibroblasts in a 3:1 ratio. Fibroblasts were seeded 1.5 × 10^5^ cells per well individually or in combination with tumor cells. Cells were left overnight to attach. The following day, cells were washed 3 times with PBS and 600-700 μL serum-free media was added to each well. After incubation for 48 or 72 hours (depending on experiment) whole-cell lysates or RNA were collected and analyzed by immunoblotting or qRT-PCR.

### qPCR analysis

Cells were seeded in media containing 10% serum which was changed to 5% serum or serum-free media the following day. RNA extraction was performed using the TRIzol Reagent (Cat# 15596-026; Invitrogen) according to the manufacturer’s instructions. cDNA samples were prepared from equal amounts of RNA using M-MLV RT (Cat# M1701; Promega; Madison, WI), and then amplified using 5X HOT FIREPol EvaGreen qPCR Mix Plus (ROX) (Cat# 08-24-00001; Solis Biodyne, Tartu, Estonia) in the Applied Biosystems StepOnePlus Real-Time PCR System (Thermo Fisher Scientific; Waltham, MA). Samples were run in triplicate. Results were analyzed as follows: threshold cycle (Ct) values were normalized using the mean Ct for the reference gene, 5SrRNA, defined as ΔCt = Ct (test gene) – Ct (mean for the reference gene). The final data were presented as the fold change (FC) between the test and control samples, defined as FC = 2^-(ΔCt (test gene) – ΔCt (mean for control)). In the co-culture experiment analysis, for a more accurate comparison to the co-culture, individually cultured epithelial cell and fibroblast cell values were combined in a weighted 3:1 ratio, defined as ΔCt (3:1) = ΔCt (epithelial cells)*0.75 + ΔCt (fibroblast cells)*0.25. These ΔCt (3:1) values were then considered the control values for comparison to the ΔCt (co-culture) test values. Final data were presented as the FC as defined above. Human primer sequences are as follows: VEGFA, (Forward: ATCCTGTGTGCCCCTGATGC, Reverse: ATGTGCTGGCCTTGGTGAGG); HBEGF, (Forward: TGTGGTGCTGTCATCTGTCTG, Reverse: AGCACAAGTCTCTCTCAGTGG); IL8, (Forward: TACTCCAAACCTTTCCACCC, Reverse: AAAACTTCTCCACAACCCTC); IL6, (Forward: CCAGGAGCCCAGCTATGAAC, Reverse: CCCAGGGAGAAGGCAACTG); IL1B, (Forward: ATGATGGCTTATTACAGTGGCAA, Reverse: GTCGGAGATTCGTAGCTGGA); TNFA (Forward: GAGGCCAAGCCCTGGTATG, Reverse: CGGGCCGATTGATCTCAGC); and 5SrRNA (Forward: GGCCATACCACCCTGAACGC, Reverse: AGCCTACAGCACCCGGTATT).

### Statistical analysis

Data in each experiment was compared using the Student’s *t* test. Statistical significance was achieved when *P*<0.05.

## SUPPLEMENTARY MATERIALS FIGURES



## Abbreviations

TAFs: Tumor-associated fibroblasts
HSP27: heat-shock protein 27
MAPK: mitogen-activated protein kinase
FN1: Fibronectin
VEGFA: vascular endothelial growth factor A
HBEGF: heparin binding EGF like growth factor
TGF-β: transforming growth factor beta
TAK1: TGF-beta activated kinase 1
TNF: Tumor necrosis factor
MMP9: matrix metalloproteinase 9
