# Supplementary material for: Tumor p38MAPK signaling enhances breast carcinoma vascularization and growth by promoting expression and deposition of pro-tumorigenic factors
Source: Oncotarget. 2017 Jun 28;8(37):61969–81. doi: 10.18632/oncotarget.18755 (PMC5617479; doi:10.18632/oncotarget.18755)
Supplement: Supplementary file 1 [file oncotarget-08-61969-s001.pdf]

Tumor p38MAPK signaling enhances breast carcinoma vascularization and growth by promoting expression and deposition of pro-tumorigenic factors

SUPPLEMENTARY FIGURES

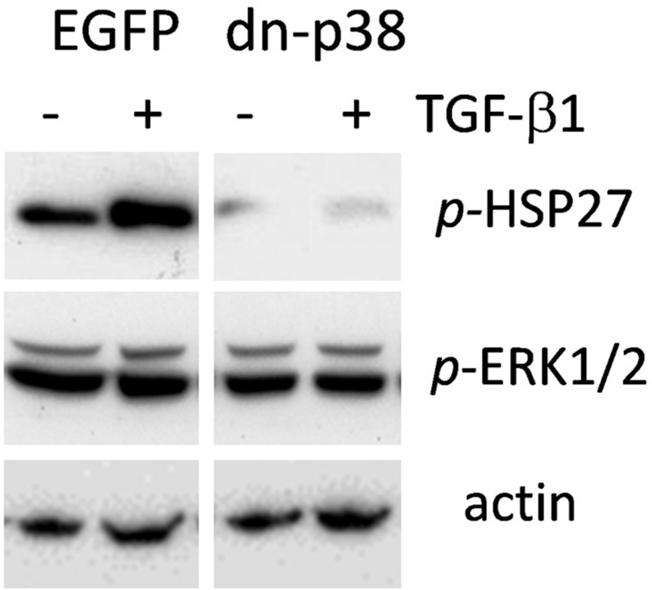

Supplementary Figure 1: Immunoblots of phospho-HSP27, phospho-ERK and beta-actin in lysates from control-EGFP and dn-p38 MDA-MB-231 cells treated with 2 ng/mL TGF-β1 for 2 hours.

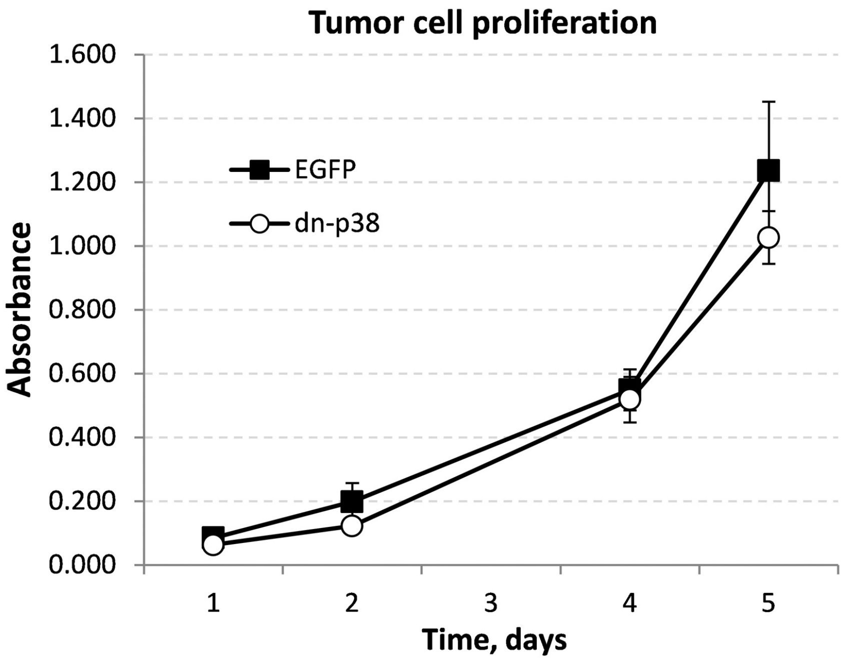

Supplementary Figure 2: Growth of control-EGFP and dn-p38 MDA-MB-231 cells in media containing 5% serum for indicated time.

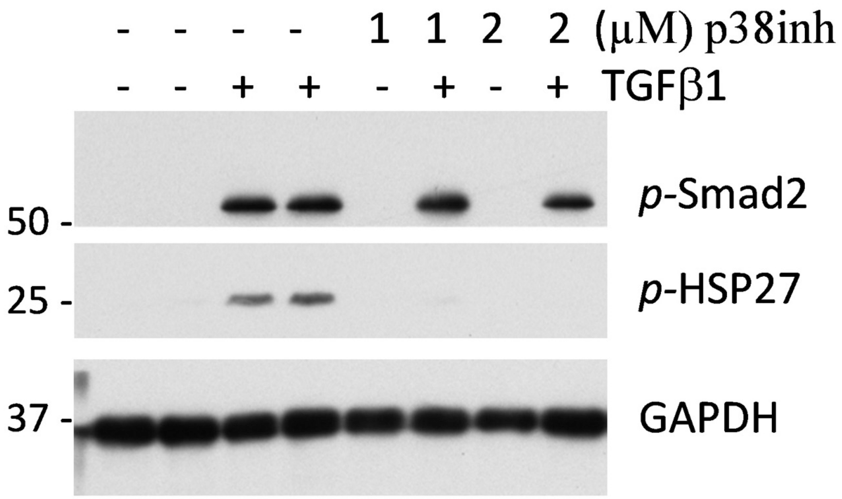

Supplementary Figure 3: Assessment of the effect of p38 inhibitor on TGF-β1 signaling. Immunoblots of phospho-SMAD2, phospho-HSP27 and GAPDH in lysates from MDA-MB-231 cells treated for 2 hours with 2 ng/mL TGF-β1 ± 1-2μM SB202190, a p38MAPK inhibitor.

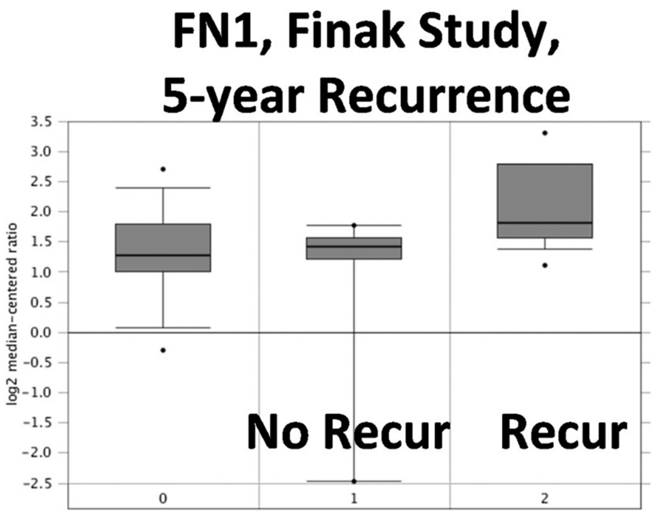

Supplementary Figure 4: Relapse-free status and mRNA levels of fibronectin in the breast cancer stroma. The data are obtained using Oncomine tools (www.oncomine.org) and the Finak Breast dataset (*Nature Medicine*, 2008). The Finak study assesses gene expression in the stroma adjacent to invasive breast carcinomas and normal tissues. The data show that a 5-year disease-free status inversely correlates with elevated mRNA levels of Fibronectin.
